# The Association of HMGB1 Expression with Clinicopathological Significance and Prognosis in Hepatocellular Carcinoma: A Meta-Analysis and Literature Review

**DOI:** 10.1371/journal.pone.0110626

**Published:** 2014-10-30

**Authors:** Lu Zhang, Jianjun Han, Huiyong Wu, Xiaohong Liang, Jianxin Zhang, Jian Li, Li Xie, Yinfa Xie, Xiugui Sheng, Jinming Yu

**Affiliations:** 1 Department of Gynecologic Oncology, Shandong Cancer Hospital and Institute, School of Medicine and life Science, University of Jinan-Shandong Academy of Medical Science, Jinan, Shandong, P.R. China; 2 Department of Cancer Interventional Radiology, Shandong Cancer Hospital and Institute, Jinan, Shandong, P.R. China; 3 Department of Cancer Interventional Radiology, Shandong Cancer Hospital and Institute, Jinan, Shandong, P.R. China; 4 Department of Immunology, Shandong University School of Medicine, Jinan, Shandong, China; 5 Department of Cancer Interventional Radiology, Shandong Cancer Hospital and Institute, Jinan, Shandong, P.R. China; 6 Department of Cancer Interventional Radiology, Shandong Cancer Hospital and Institute, Jinan, Shandong, P.R. China; 7 Provincial Key Laboratory of Radiation Oncology, Shandong Cancer Hospital and Institute, Jinan, Shandong, P.R. China; 8 Department of Cancer Interventional Radiology, Shandong Cancer Hospital and Institute, Jinan, Shandong, P.R. China; 9 Department of Gynecologic Oncology, Shandong Cancer Hospital and Institute, Jinan, Shandong, P.R. China; 10 Department of Radiation Oncology, Shandong Cancer Hospital and Institute, Jinan, Shandong, P.R. China; University of Modena & Reggio Emilia, Italy

## Abstract

**Background:**

Hepatocellular carcinoma (HCC) is the fifth most common cancer, and it is the second most common cancer-related mortality globally. The prognostic value of high mobility group box 1 (HMGB1) remains controversial. The purpose of this study is to conduct a meta-analysis and literature review to evaluate the association of HMGB1 expression with the prognosis of patients with HCC.

**Methods:**

A detailed literature search was made in Medline, Google Scholar and others for related research publications. The data were extracted and assessed by two reviewers independently. Analysis of pooled data were performed, Hazard Ratio (HR) and mean difference with corresponding confidence intervals (CIs) were calculated and summarized respectively.

**Results:**

10 relevant articles were included for this meta-analysis study. HMGB1 mRNA levels in HCC were significantly higher than those in normal (p<0.00001) and para-tumor tissues (p = 0.002) respectively. The protein levels of HMGB1 in HCC were significantly higher than those in para-tumor tissues (p = 0.005). Two studies reported the serum HMGB1 levels in patients with HCC of TNM stages, and indicating significantly different between stage I and II, stage II and III, as well as stage III and IV (two studies showed p<0.01 and p<0.001 respectively). The overall survival (OS) was significantly shorter in HCC patients with high HMGB1 expression compared those with low HMGB1 expression and the pooled HR was 1.31 with 95% CI 1.20–1.44, Z = 5.82, p<0.0001. Two additional studies showed that there were higher serum HMGB1 levels in patients with chronic hepatitis than those in healthy people (p<0.05).

**Conclusions:**

The results of this meta-analysis suggest that HMGB1 mRNA and protein tissue levels in the patients with HCC are significantly higher than those in para-tumor and normal liver tissues respectively. Tissue HMGB1 overexpression is a potential biomarker for HCC diagnosis, and it is significantly associated with the prognosis of patients with HCC.

## Introduction

Hepatocellular carcinoma (HCC) is the fifth most common cancer in men, worldwide, and seventh among women, and it is the second leading cause of cancer-related mortality globally [1]. HCC commonly occurs in Asia and Africa, and its incidence rate is starting to increase in Western countries [2], [3]. Due to lack of effective biomarkers for diagnosis and prognosis, HCC is frequently found in late stages, when curative therapy approaches like resection, liver transplantation, radio frequency ablation (RFA), and transarterial chemoembolization (TACE) don't produce satisfactory clinical outcomes. HCC patients present with high recurrence and metastasis [4], [5], the 5-year survival rate after surgery for HCC has been only 25%–39% [6]. Therefore, it is critical to identify the biomolecular markers for the diagnosis and prognosis to monitor recurrence of HCC.

The serum a-fetoprotein (AFP) has been widely used as a risk assessment factor in patients with cirrhosis and a screening method for early detection of HCC patients, and a prognostic factor for prediction of tumor recurrence. AFP is the only HCC biomarker that has been studied through to phase 5 of biomarker development [7]. However, its sensitivity and specificity vary significantly ranging from 40%–65% and 76%–96%, respectively [8], [9], [10]. Recent studies have identified other potential biomarkers for early detection of HCC, including the circulating AFP isoform AFP-L3 [11], [12], des-gamma-carboxy prothrombin (DCP) [12], Golgi protein-73 (GP73) [13] and circulating miRNA [14]. However, most of them are only in phase 1 or 2 stages, further studies are needed to determine whether these biomarkers can be translated from bench works to clinical settings. The effects of other biomarkers to predict the prognosis for HCC such as vascular endothelial growth factor (VEGF), Cyclin-dependent Kinases(CDK), β-catenin/Wnt pathway and microRNAs [15] have been controversial and again none of them has been applied to the clinical settings.

HMGB1 was discovered in calf thymus in 1973 and named for its electrophoretic mobility in polyacrylamide gels [16], [17]. HMGB1 is encoded on human chromosome 13q12-13 and consists of 215 amino acids. It is a highly conserved protein containing two DNA binding domains A and B as well as a negatively charged C-terminal tail domain [18], [19]. HMGB1 has different functions depending on its subcellular locations. HMGB1 is normally located in the nucleus where it acts as a DNA chaperon by regulating transcription, replication, recombination, repair, and genome stability [20]. In 2011, Hanahan and Weinberg proposed a new model to define the ten hallmarks for the tumor requirements [21]. Recent findings indicate that HMGB1 dysfunction is associated with every hallmark of cancer and contributes to cancer initiation and development [22]. HMGB1 overexpression and cytoplasmic localization have been observed in some cancer cells such as in colon cancer [23], gastrointestinal stromal tumors [24], cervical carcinomas [25] and melanoma [26]. Moreover, recent studies demonstrate that HMGB1 expression is a useful prognostic maker for colorectal cancer and diffuse malignant peritoneal mesothelioma [27], [28], [29]. Higher HMGB1 expression has been observed in HCC compared to normal liver tissues [30], [31], [32]. HMGB1 could be a potential biomarker that may be used to detect HCC at early stage and predict its prognosis. However, the prognostic value of HMGB1 for survival remains controversial due to limited statistic power. There is no meta-analysis study to reveal the association between HMGB1 expression and clinicopathological parameters of HCC. Therefore, we performed a meta-analysis to evaluate the association of HMGB1 expression with the clinicopathological parameters and prognosis in HCC.

## Methods

### Search strategy and selection criteria

The following electronic databases were searched for relevant articles without any language restrictions: Web of Science (1945 ∼ 2014), the Cochrane Library Database (Issue 12, 2014), PubMed (1966 ∼ 2014), EMBASE (1980 ∼ 2014), CINAHL (1982 ∼ 2014), CNKI, Google scholar, and the Chinese Biomedical Database (CBM) (1982 ∼ 2014). We searched articles using the following terms: HMGB1 or high motility group box 1 and hepatocellular carcinoma, liver cancer or HCC. There were 27 articles identified from PubMed, 31 articles from Web Science, 33 articles from EMBASE, 262 articles from CNKI. 5950 articles from Google scholar were identified, however, only 500 of them were screened by titles and abstracts since the rest of them were not relevant. Finally total 853 articles were screened by titles and abstracts.

**Figure 1:**
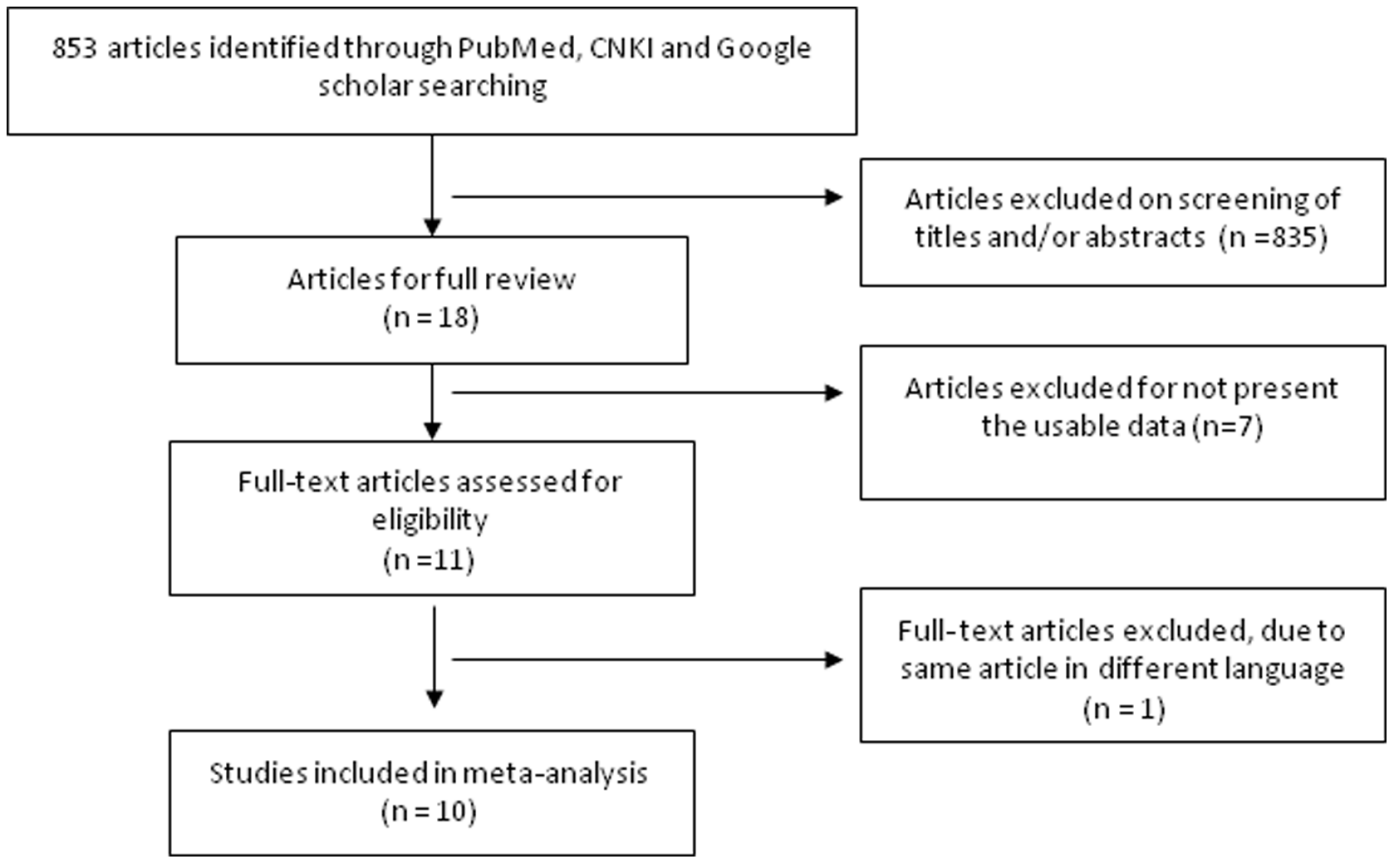
Schematic flow diagram for selection of included studies.

The inclusion criteria included: 1) The articles which the association between HMGB1 expression and the clinicopathological significance of HCC was evaluated; 2) The articles which the association of HMGB1 expression and prognosis in patients with HCC was evaluated; 3) The studies which utilized RT-PCR for detection of HMGB1 mRNA, ELISA for serum HMGB1 and Western Blot for tissue HMGB1 expression. The exclusion criteria included: 1) The studies which used the same population or overlapping database; 2) The studies of in vitro cell culture models (Figure 1). The search identified 18 articles of which 10 were eligible for quantitative analysis in this meta-analysis. The detailed information of 10 relevant citations was listed in Table 1.

**Table 1:** Main Characteristics of included studies.

Author	Year	HCC Sample size	Comments
Liu et al. [31]	2012	161	High expression of HMGB1 predicts poor prognosis for HCC
Jiang et al. [30]	2012	34	HMGB1 is associated with clinicopathologic features in patients with HCC
Cheng et al. [70]	2008	161	Serum HMGB1 is associated with clinicopathologic features in patients with HCC
Xiao et al. [56]	2014	208	The association of HMGB1 Gene with the prognosis of HCC
Ueno et al. [32]	2011	36	Significance of HMGB1 expression in HCC
Han et al. [71]	2014	13	Changes of HMGB1 expression in HCC
Gou et al. [72]	2013	82	Detection and clinical significance of HMGB1 in HCC
Wang et al. [73]	2013	39	Increased HMGB1 expression in HCC and its mechanism
Feng et al. [74]	2011	40	The expression and clinical significance of HMGB1 in HCC
Wang et al. [57]	2013	155	Expression, subcellular location and prognostic relevance of HMGB1 in HCC

### Data extraction and study assessment

Two reviewers (Zhang and Han) independently extracted data and reviewed the contents of the manuscripts to determine whether they met the criteria for inclusion. Any discontent was discussed and resolved by a consensus including a third investigator (Wu). A data extract form was developed accordingly. One review author (Zhang) extracted the following data from the included studies: first author's name, year of publication, number of patients, stage of HCC, grade of HCC, and HMGB1 expression. The second author (Han) checked the extracted data, and disagreement was resolved by the discussion with the third author (Wu) for all issues.

### Statistics analysis

All analysis was performed with Review Manager 5.2. Heterogeneity between studies was assessed using the Q-test and *I* ^2^ index. Mean differences with 95% confidence intervals were calculated by using a fixed or random effect model depending on heterogeneity (a fixed effect model for *I* ^2^<50%, a random effect model for *I* ^2^>50%). Meta-analysis was performed to compare HMGB1 mRNA expression between HCC and normal tissue, HMGB1 mRNA and protein expression levels between HCC and para-tumor tissue. Two studies investigated serum HMGB1 protein levels in TNM stages of HCC that were listed in Table 2. Another two studies detected serum HMGB1 levels in patients with hepatitis and normal controls, the data were listed in Table 3. The multivariate HRs were collected and the log HRs and its standard errors were calculated for individual study. Pooled hazard ratio (HR) with a 95% confidence interval was calculated for the association between HMGB1 expression and prognosis. All p values were two sided. Funnel plots were used for detection of publication bias. A sensitivity analysis, in which one study was removed at a time, was conducted to assess the result stability.

**Table 2:** Serum HMGB1 levels (ng/ml) in TNM stages of patients with HCC.

Author	Sample size	I	II	III	IV	p	Detect method
Gou [72]	82	29.1±5.0	57.7±14.2	101.6±14.8	176.0±32.5	<0.01	Elisa
Cheng [70]	161	30.2±5.9	58.9±20.0	100.9±23.4	175.8±47.6	<0.0001	Western-blot

**Table 3:** Serum HMGB1 levels (ng/ml) in patients with hepatitis and normal controls.

Author	Sample size (Heptitis/Normal)	Chronic Hepatitis	Normal control	P	Detect method
Wang [73]	39/48	13.5±6.3	3.9±1.4	<0.01	Elisa
Feng [74]	38/23	48.6±18.38	36.6±7.07	<0.05	Elisa

## Results

Total 853 articles were screened by titles and abstracts. The search identified 18 articles of which 10 were eligible for quantitative analysis in this meta-analysis. 7 articles were excluded due to different detect methods used. One article was excluded due to duplication. 10 relevant articles were included for full review in detail and 6 of them were conducted with meta-analysis (Figure 1). The following items were collected from each study: first author's name, year of publication, number of patients, stages of HCC, detect methods, HMGB1 expression and prognosis (Table 1 and 2).

**Figure 2:**
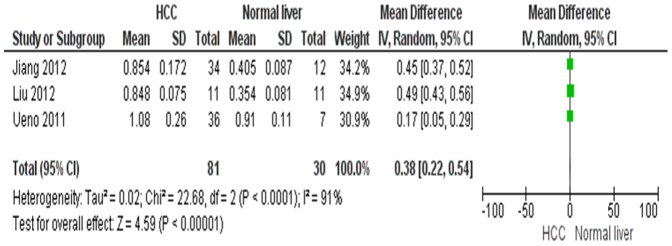
Forest plot for HMGB1mRNA/GADPH in HCC and normal liver tissue.

**Figure 3:**
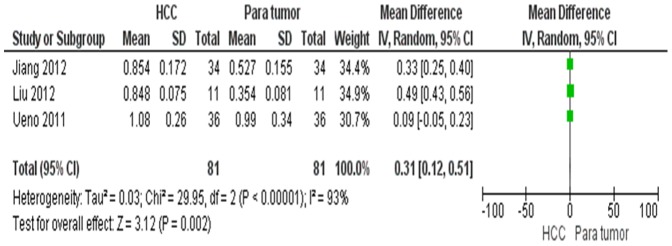
Forest plot for HMGB1mRNA/GADPH in HCC and para-tumor tissue.

**Figure 4:**
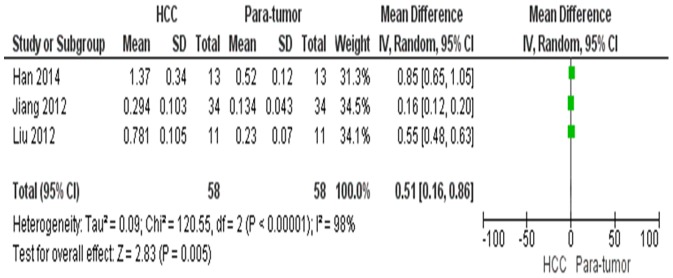
Forest plot for HMGB1 protein/GADPH in HCC and para-tumor tissue.

**Figure 5:**
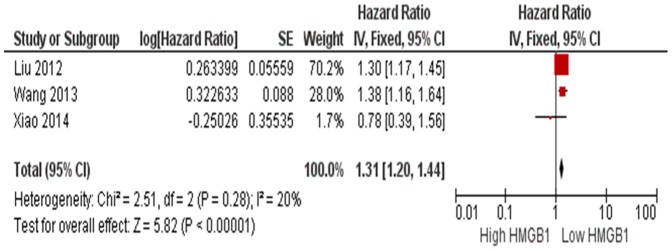
Forest plot for the association of HMGB1expression level and the risk of HCC

HMGB1 mRNA level in HCC was significantly higher than in normal and para-tumor tissues respectively. The pooled mean difference for HCC versus normal liver tissue was 0.38, (95% CI 0.22–0.54, Z = 4.59, p<0.00001, heterogeneity *I* ^2^ = 91%, Figure 2) and for HCC versus para-tumor, mean difference was 0.31, (95% CI 0.12–0.51, Z = 3.12, p = 0.002, heterogeneity *I* ^2^  = 93%, Figure 3). The protein level of HMGB1 in HCC was significantly higher than those in para-tumor tissues, the pooled mean difference was 0.51 with 95% CI 0.16–0.86, (Z = 2.83, p = 0.005, heterogeneity *I* ^2^ = 98%, Figure 4). Figure 5 showed the result of a meta-analysis of hazard ratios (HR) of HMGB1 high expression and low expression in the survival of patients with HCC. The overall survival (OS) was significantly shorter in HCC patients with high HMGB1 expression compared those with low HMGB1 expression, and the pooled HR was 1.31 with 95% CI 1.20–1.44, (Z = 5.82, p<0.0001, heterogeneity *I* ^2^ = 20%, Figure 5).

Four of included studies were fully reviewed without performing meta-analysis due to less number of patients in the selected articles. Two studies reported the serum HMGB1 levels in patients with HCC of TNM stage I, II, III, IV (Table 2). The differences were significant between stage I and II, stage II and III, as well as stage III and IV groups (two studies showed p<0.01 and p<0.0001 respectively). Another two studies investigated the serum HMGB1 levels, suggesting there were higher serum HMGB1 levels in patients with chronic hepatitis than in healthy people (p<0.01 and p<0.05 respectively) (Table 3).

**Figure 6:**
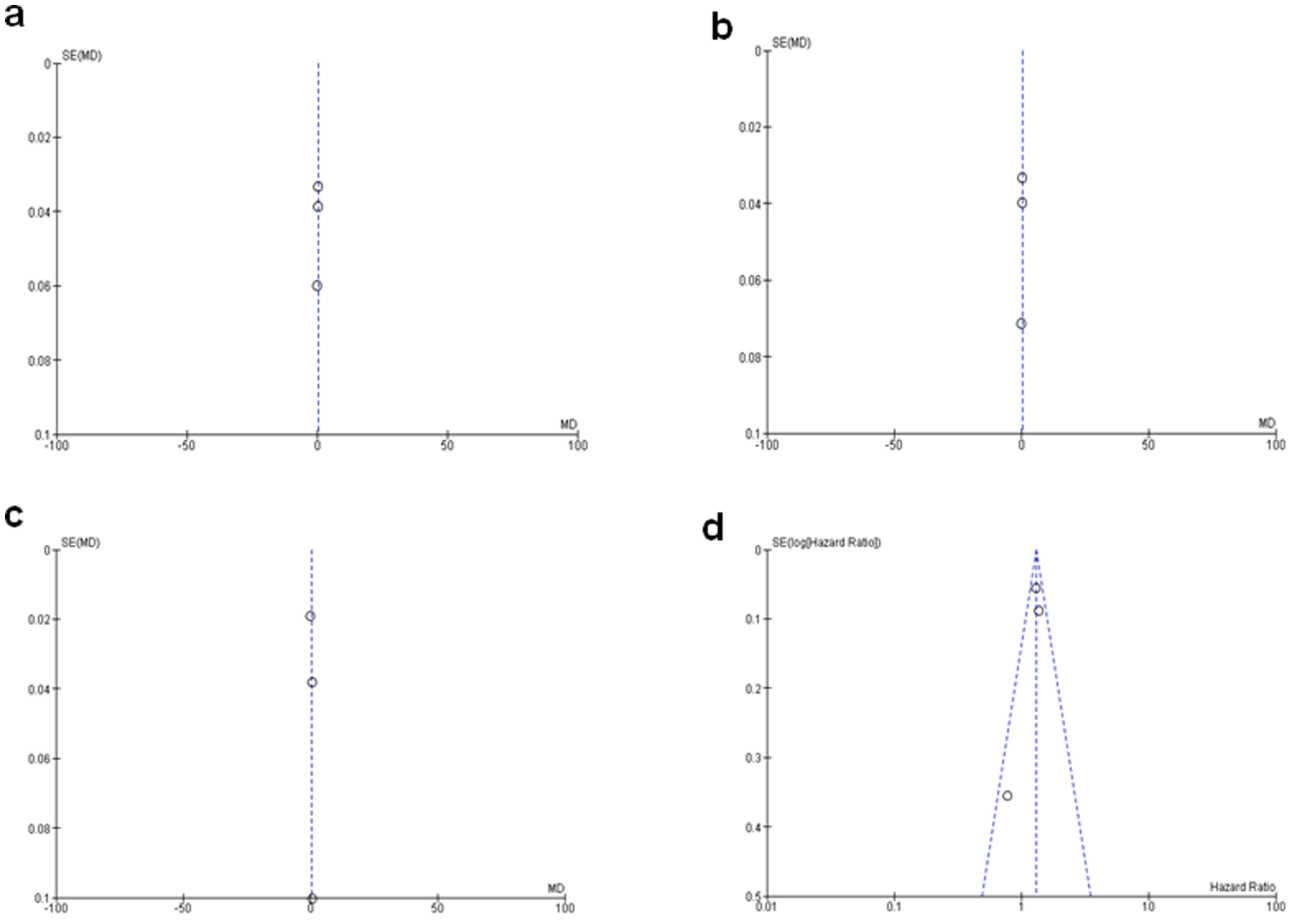
Funnel plot for publication bias. a: HMGB1 mRNA/GADPH in HCC and normal liver tissue; b: HMGB1 mRNA/GADPH in HCC and para-tumor tissue; c: HMGB1 protein/GADPH in HCC and para-tumor tissue; d: the association of HMGB1expression and the risk of HCC.

A sensitivity analysis, in which one study was removed at a time, was conducted to assess the result stability. The pooled HR was not significantly changed, indicating the stability of our analyses. The funnel plots were largely symmetric (Figure 6) suggesting there were no publication biases in the meta-analysis of HMGB1 expression and clinicopathological features.

## Discussion

For the first time, we conducted a meta-analysis and evaluated HMGB1 mRNA and protein expression levels in tissues from patients with HCC and normal liver or para-tumor tissues. The heterogeneity between studies was assessed using the Q-test and *I* ^2^ index. All *I* ^2^ indexes for comparisons of mRNA and protein expression between HCC and normal or para-tumor tissue were great than 75%, indicating that there was significant heterogeneity between studies. All p values for test of overall effect were equal or less than 0.005, indicating mRNA and protein expression levels in HCC were significantly increased compared to those in the normal and para-tumor tissues respectively. This suggests that HMGB1 plays an important role in the pathogenesis of HCC. HMGB1 is a potential tissue biomarker for HCC diagnosis.

Cancer development is a multistep process. Accumulated evidences indicate that HMGB1 is associated with ten functional capabilities of cancers as Hanahan and Weinberg described in 2011 [21]. Its function depends on the subcellular locations. Within the nucleus, HMGB1 acts as a DNA chaperon to bind to the minor groove of DNA, stabilizing nucleosides and facilitating the assembly of site-specific DNA-binding proteins such as the nuclear hormone/nuclear hormone receptor complex and p53, p73, as well as retinoblastoma protein (Rb) transcription complexes [33], [34]. When HMGB1 releases outside of the cell, it interacts with a wide range of proteins, such as the receptor of advanced glycation end products (RAGE), Toll-like receptors (TLRs: TLR2, TLR4 and TLR9), mitogen-activated protein kinase (MAPK), NF (nuclear factor)-κB, and coordinates immune system activation, cell migration, cell growth, angiogenesis, and tissue repair as well as regeneration [35], [36], [37], [38], [39], [40].

Recent studies report that the expression of HMGB1 is upregulated in several types of malignancies including gastric cancer [41], breast cancer [33], nasopharyngeal carcinoma [42], and squamous cell carcinoma of the head and neck [43], [44]. HMGB1 is not only constitutively expressed in the nucleus of cancer cells, but also is released by the tumor cells, macrophages, monocytes, dendritic cells (DCs), and T cells [45]. Released HMGB1 that acts as a proinflammatory cytokine from necrotic tumor cells creates a microenvironment and triggers signaling pathway, such as the NF-κB and inflammasome, to induce proinflammatory cytokine release. This loop accelerates inflammatory response and tumor formation [22], [46], [47]. Therefore, serum HMGB1 levels could be prediction for progression of patients with HCC.

Two studies reported the serum HMGB1 levels in patients with HCC of TNM stage I, II, III, IV. The differences were significant between stage I and II, stage II and III, as well as stage III and IV groups (two studies showed p<0.01 and p<0.0001 respectively). Higher HMGB1 expression was detected in advanced stages of HCC patients. This suggests that HMGB1 plays an important role in the invasion, metastasis and progression of HCC. In support of this, recent evidences indicated HMGB1 was co-localized with RAGE, suggesting their potential contribution to cellular migration and tumor invasion. The expression of RAGE was closely associated with the invasive and metastatic behavior in different types of cancer such as gastric [48] and colorectal cancer [49]. The interaction of HMGB1 with RAGE leads to activation of the NF-κB, MAPK, and type IV collagenase (MMP-2/MMP-9) signaling pathways, all of which degrade extracellular matrix protein and play a major role in tumor invasion and metastasis [50], [51], [52], [53]. Furthermore, tumor growth and metastasis were suppressed by blocking RAGE-HMGB1 complex in human and murine cancer cells [52], [54]. Knockdown of HMGB1 gene also inhibited liver cancer growth and metastasis [31], [55]. Therefore, the overexpression of HMGB1 is not only a potential biomarker for initial diagnosis of HCC, but it also could be a predictor of prognosis in patients with HCC. More studies are needed for a meta-analysis in the future to confirm the progression value of HMGB1 in the patients with HCC.

A few of studies reported controversial results of the association between HMGB1 expression and the prognosis of patients with HCC [31], [56], [57]. Those studies on prognostic significance of HMGB1 expression were limited by low statistical power. We performed a meta-analysis for the first time and pooled three studies together (n = 524). The low heterogeneity (*I* ^2^ = 20%) could be due to different methods to measure HMGB1 intensity in immunohistochemistry. Our pooled HR was 1.31 with 95% CI 1.20–1.44, test for overall effect p<0.0001, suggesting that high HMGB1 expression was significantly associated with the mortality of patients with HCC. Compared with low HMGB1 expression, HMGB1 high expression was associated with more than 1.3 times the risk of mortality. HMGB1 affects the prognosis of HCC through complicated pathways. HMGB1 has the following abilities to induce tumorigenesis: 1). tumor cells and tumor-infiltrating leukocytes secret HMGB1 in tumor microenvironment which activates NK-κB and inflammatory pathways, promoting tumor growth, invasion and metastasis [46], [47], [58]; 2). HMGB1 also has the ability to increase the production of ATP and provide more energy for tumor growth [59]; 3). In addition, HMGB1 promotes angiogenesis [60], [61], [62], [63] for tumor growth and metastasis; 4). Lastly, HMGB1 inhibits antitumor immunity for tumor survival [64], [65], [66]. All these activities contribute to poor prognosis of HCC patients with high expression of HMGB1. Therefore, HMGB1 overexpression predicts a poor prognosis in patients with HCC.

Interestingly, two studies investigated serum HMGB1 in patients with chronic hepatitis (the precursor of HCC) and healthy people. Significantly higher serum HMGB1 was detected in the patients with chronic hepatitis compared to those detected in healthy people. The difference of serum HMGB1 levels between the two studies could be due to that different methods were used to detect the serum concentration of HMGB1. HMGB1 lacks a classic signal sequence. During infection, HMGB1 is translocated from the nucleus to the cytoplasm and is subsequently released into the extracellular milieu by HCV or HBV infection [67], [68], [69]. HMGB1 is likely to be crucial in initiating and mediating hepatitis. Further studies are needed to verify its role in the pathogenesis of chronic hepatitis and subsequently in HCC.

We evaluated the HMGB1 expression in patients with HCC (both tumor and para-tumor tissues) and normal liver tissues. Further studies should be focused on finding cut-offs for the diagnosis of HCC and determination of its stages. In the future, more studies are needed to verify if HMGB1 blockage can be used in the development of new therapeutic agents for HCC. Finally, our study only selected the published articles, but it did not include some relevant unpublished papers which may result in certain publication bias. Thus the result should be interpreted carefully.

In conclusion, HMGB1 mRNA and protein tissue levels in the patients with HCC are significantly higher than in those of para-tumor and normal liver tissues respectively. HMGB1 overexpression is a potential biomarker for HCC diagnosis, and it significantly associated with the prognosis of patients with HCC.

## Supporting Information

Checklist S1 **A PRISMA Checklist.**
(DOC)
